# Conformational Plasticity of Centrin 1 from *Toxoplasma gondii* in Binding to the Centrosomal Protein SFI1

**DOI:** 10.3390/biom12081115

**Published:** 2022-08-13

**Authors:** Luca Bombardi, Filippo Favretto, Marco Pedretti, Carolina Conter, Paola Dominici, Alessandra Astegno

**Affiliations:** Department of Biotechnology, University of Verona, Strada Le Grazie 15, 37134 Verona, Italy

**Keywords:** centrin, SFI1 protein, *Toxoplasma gondii*, calcium, protein-peptide interactions

## Abstract

Centrins are calcium (Ca^2+^)-binding proteins that are involved in many cellular functions including centrosome regulation. A known cellular target of centrins is SFI1, a large centrosomal protein containing multiple repeats that represent centrin-binding motifs. Recently, a protein homologous to yeast and mammalian SFI1, denominated TgSFI1, which shares SFI1-repeat organization, was shown to colocalize at centrosomes with centrin 1 from *Toxoplasma gondii* (TgCEN1). However, the molecular details of the interaction between TgCEN1 and TgSFI1 remain largely unknown. Herein, combining different biophysical methods, including isothermal titration calorimetry, nuclear magnetic resonance, circular dichroism, and fluorescence spectroscopy, we determined the binding properties of TgCEN1 and its individual N- and C-terminal domains to synthetic peptides derived from distinct repeats of TgSFI1. Overall, our data indicate that the repeats in TgSFI1 constitute binding sites for TgCEN1, but the binding modes of TgCEN1 to the repeats differ appreciably in terms of binding affinity, Ca^2+^ sensitivity, and lobe-specific interaction. These results suggest that TgCEN1 displays remarkable conformational plasticity, allowing for the distinct repeats in TgSFI1 to possess precise modes of TgCEN1 binding and regulation during Ca^2+^ sensing, which appears to be crucial for the dynamic association of TgCEN1 with TgSFI1 in the centrosome architecture.

## 1. Introduction

Centrins are small calcium (Ca^2+^)-binding proteins that are highly conserved throughout the eukaryote kingdom and are frequently associated with centrosome-related structures such as centrioles and basal bodies, with key functions in their duplication [1,2,3]. There is increasing evidence that centrins are not restricted to centrosomes, but are found at several other sites in the cell, consistent with their emerging activities in several cellular processes, including DNA repair, mRNA nuclear export, and signal transduction [1,4,5,6,7,8,9,10]. The specificity of localization and functional diversity of centrins is likely achieved through interaction with multiple binding partners at centrosomes and spindle pole bodies (SPBs), or in other cellular compartments or organelles [4,5,6,7,8,11,12,13,14,15,16,17].

Centrins are closely related to calmodulin (CaM) and, similar to CaM, possess four helix-loop-helix motifs (EF-hand) for Ca^2+^ binding that fold into two domains (or lobes), the N- and C-terminal domains, which are connected by a flexible α-helical region. Binding of Ca^2+^ to centrins likely results in conformational changes from closed to open form, exposing hydrophobic regions that allow the protein to bind to various target proteins. Structural studies of centrin interactions have revealed that the basis for the target selectivity of centrins lies in their ability to interact with regions that have the propensity to form an α-helix and contain a hydrophobic triad, namely W^1^xxL^4^xxxL^8^ (1–4–8 motif), which can be either in a forward (W^1^L^4^L^8^) or reverse (L^8^L^4^W^1^) orientation [6,12,18]. This sequence has been found in many centrin–target complex structures and validated by various biophysical techniques [6,12,17,18,19,20,21,22,23].

Three different centrins are expressed in *Toxoplasma gondii*, a protozoan parasite that causes toxoplasmosis [24]. Similar to other canonical centrins, centrins 1 and 3 from *T. gondii* (TgCEN1 and TgCEN3) are mainly localized to the centrioles, while TgCEN2 is found at the centrosome, peripheral annuli, preconoid ring, and basal cup of the parasites, where it mediates various aspects of the parasite’s life cycle [25,26]. However, the precise functions of centrins and their physiological targets in Toxoplasma remain unclear.

A known binding partner of centrins in yeast and mammalian cells is SFI1 (suppressor of fermentation-induced loss of stress resistance 1) [13]. SFI1 is a large, α-helical protein first discovered in yeast, where it promotes SPB duplication by interacting with the yeast CDC31 centrin [13,27]. The human homolog of SFI1 is localized to the centrosome and binds the centrin 2 molecule (HsCEN2) [12,13]. Recently, SFI1 was suggested to promote centriole duplication in human cells, similar to its function in yeast SPBs [28]. A common feature of the SFI1 sequence is the presence of multiple (~20) continuous internal repeats with the consensus motif AX7L**L**X3**F/L**X2**W**K/R (in bold, the hydrophobic triad) [12,27], which represent centrin-binding motifs. This suggests a general model for the centrin–SFI1 complex, in which many centrins are bound to the different repeats of a single SFI1 molecule, likely resulting in a filamentous structure. Crystallographic studies of yeast CDC31 bound to two or three repeats of yeast SFI1 have shown that the centrin molecules wrap around the long α-helix of SFI1 [27] independently of Ca^2+^. Interestingly, centrins were found to interact not only with SFI1, but also with each other; in particular, the C-terminal domain of one centrin was found to interact with the N-terminal domain of the next centrin, supporting the formation of a centrin filament using the long α-helix of SFI1 as a scaffold.

Recently, an ortholog of human and yeast SFI1, designated TgSFI1, was identified in *T. gondii*, which has multiple conserved centrin-binding repeat sequences [29]. TgSFI1 was found to be tightly colocalized with TgCEN1 in the outer core of the parasite centrosome [29]. Notably, a temperature-sensitive mutant of *T. gondii* (ts-TgSFI1) carrying a point mutation in the gene coding for TgSFI1 displays severe failure in cell division and absence of budding [29,30]. Furthermore, in accordance with the role of SFI1 as a known centrosome partner of centrin, the loss of ts-TgSFI1 is accompanied by a significant loss of TgCEN1-associated cores at high temperatures [29].

Our previous work has shown that TgCEN1 has biochemical properties consistent with a role as a Ca^2+^ sensor, since it is able to expose hydrophobic surfaces for interaction with signaling partners upon Ca^2+^ binding [19,31]. Thus, its cellular role should be mediated by binding to target proteins.

Here, we used various biophysical techniques to characterize the binding of TgCEN1 to synthetic peptides corresponding to different repeats of the TgSFI1 protein. The peptides were selected considering the conservation of the centrin-binding sequences in TgSFI1, focusing on the hydrophobic triad. Our data suggest different binding modes of TgCEN1 with the isolated TgSFI1 repeats in terms of lobe-specific contribution to binding, binding affinity, and Ca^2+^ dependence. Even if it remains elusive how TgCEN1 may exert its role as a signal transducer, the diversity among its binding sites in TgSFI1 may contribute to its functional plasticity and regulatory role in the structural organization and dynamics of centrosomes and basal bodies.

## 2. Materials and Methods

### 2.1. Materials

All chemicals, unless otherwise stated, were purchased from Sigma Aldrich (St. Louis, MO, USA). All purchased chemicals were of the highest commercially available purity grade.

### 2.2. Protein Production and Peptide Synthesis

The recombinant TgCEN1 (lacking the first 21 residues) and its N-terminal (N-TgCEN1, 22–94 aa) and C-terminal (C-TgCEN1, 95–169 aa) domains were purified as previously described [19,31]. The TgSFI1 peptides were synthesized by GenScript U.S.A. Inc. (Piscataway, NJ, USA). The concentration of each peptide was determined using their predicted molar extinction coefficient at 280 nm.

### 2.3. Size Exclusion Chromatography

The Stokes radius of Ca^2+^-bound TgCEN1, alone and in complex with selected peptides, was estimated by size exclusion chromatography (SEC) using a Superose 12 10/300 GL column (GE Healthcare) in 50 mM Tris-HCl, 150 mM KCl, 5 mM CaCl_2_, 0.5 mM DTT, pH 7.5, as described in [32,33,34]. Each experiment was performed in triplicate, and reported values represent means ± SEM.

### 2.4. Isothermal Titration Calorimetry Experiments

Isothermal titration calorimetry (ITC) experiments were performed on a MicroCal PEAQ-ITC instrument (Malvern Ltd., Malvern, UK) at 25 °C. Protein samples and peptides were solubilized in the same buffer containing 50 mM Tris–HCl, 150 mM KCl pH 7.5 and 5 mM CaCl_2_ or 5 mM EGTA. All the samples were filtered and degassed before use, and the pH was carefully determined to exclude any possible pH-related effect on ITC experiments.

A typical ITC experiment was performed by titrating 200 μL of a 30–50 μM protein solution with a 1 or 1.5 μL injection of 0.3–0.5 mM peptide (total injections 39 or 26, respectively) with an initial delay of 120 s and keeping a time gap of 120 s between injections. A reference injection of peptide into buffer without centrin was performed and subtracted from each experiment. The resulting curves were fitted to calculate apparent dissociation constants K_d_, enthalpy changes (ΔH), and the apparent entropy change (ΔS) using the software MicroCal ITC Origin 7 Analysis Software (MicroCal, Malvern Ltd., Malvern, UK). All measurements were repeated in triplicate using at least two different protein preparations and the error was calculated as the mean standard error of the three measurements.

### 2.5. Circular Dichroism Spectroscopy

Circular dichroism (CD) measurements were performed on a Jasco J-1500 spectropolarimeter (JASCO Corporation, Tokyo, Japan) equipped with a temperature control system. Far-UV spectra were recorded between 200 (or 190) and 250 nm at 25 °C using 0.1 cm quartz cuvettes. Spectra were collected as an average of three scans with the same parameters as previously described [6,35]. Protein and peptide samples were dissolved in a buffer containing 50 mM Tris–HCl, 150 mM KCl, and 0.5 mM DTT pH 7.5, and supplemented with 5 mM CaCl_2_ or 5 mM EGTA. The spectrum of the buffer alone was subtracted from that of the sample.

### 2.6. Fluorescence Spectroscopy

Fluorescence spectra were recorded on a Jasco FP8200 spectrofluorometer (JASCO Corporation, Tokyo, Japan). The binding of TgCEN1 or its domains to the different peptides was followed by monitoring the fluorescence of the Trp residue of the peptide. Excitation was set at 295 nm, and fluorescence emission was measured from 305 to 500 nm at 25 °C using quartz cuvettes, as previously described [6,35,36]. The peptide (5 μΜ) was dissolved in 50 mM Tris–HCl, 150 mM KCl pH 7.5, 0.5 mM DTT, in the presence of 5 mM CaCl_2_ or 5 mM EGTA.

### 2.7. Nuclear Magnetic Resonance Spectroscopy

Nuclear magnetic resonance spectroscopy (NMR) experiments were performed at 298 K on a 600 MHz Bruker Avance III spectrometer, equipped with a triple resonance Prodigy cryo-probe (Bruker, Karlsruhe, Germany). NMR samples (0.4 mM) were obtained by dissolving ^15^N-TgCEN1 in 50 mM Tris–HCl, 50 mM KCl pH 7.5, 0.5 mM DTT, 5% D_2_O, and 5 mM CaCl_2_. The unlabeled peptides were added in slight molar excess. ^1^H-^15^N HSQC (heteronuclear single quantum coherence) spectra were performed using previously described pulse sequences [19] and processed using Topspin v. 3.6.2 (Bruker) and NMRpipe [37,38]. Spectra analysis was carried out using the software Ccpnmr Analysis v. 2.5.2 [39].

To examine the peptide conformational rearrangements upon binding to Ca^2+^-TgCEN1, 2D ^15^N, ^13^C filtered NOESY experiments were performed with samples containing ^15^N-labeled Ca^2+^-TgCEN1 (0.4 mM) and unlabeled peptide (mixing time 120 ms; recycle delay 1.2 s; spectral window 12 ppm in both dimensions; 256 points in the *F*_1_ dimension) using standard Bruker pulse sequences.

## 3. Results

**Figure 1:**
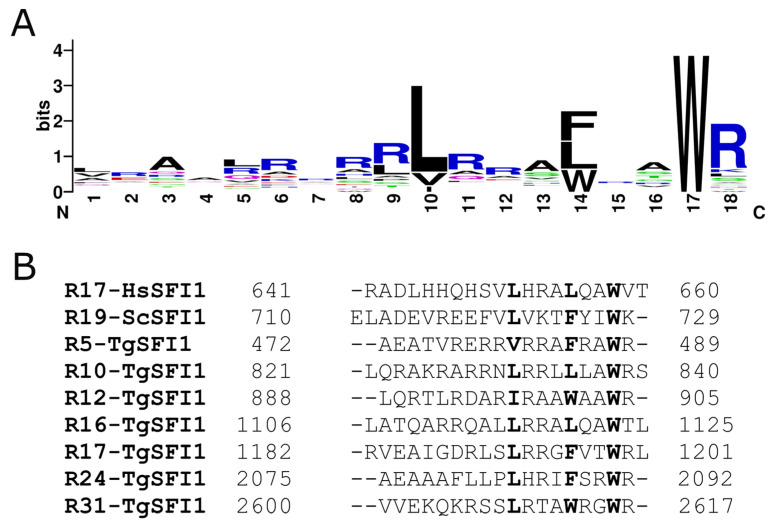
Repeat centrin-binding sequences of TgSFI1. (**A**) Sequence logo of the SFI1 repeats in *T. gondii*. The total height of all the stacked letters at a single position is proportional to the amount of conservation of the residue [41], and the height of each stacked letter is proportional to the frequency with which the amino acid is observed. (**B**) The seven peptides used in this work, localized in distinct positions in the TgSFI1 sequence, are aligned with the consensus repeat sequences of the human (HsSFI1) and yeast SFI1 (ScSFI1) proteins. The hydrophobic triad is in bold (reverse orientation).

TgSFI1 (TGME49_274000) is a large, uncharacterized protein of 3903 residues (438 kDa) that has only ~17% and ~24% sequence identity with the known yeast and human SFI1 proteins, respectively. A lack of homology exists in the N- and C-terminal regions, where TgSFI1 has large residual extensions compared to SFI1 from other organisms. Although TgSFI1 is a globally divergent protein, analysis of its sequence allowed us to identify 32 potential centrin-binding sites that resemble the internal consensus repeats in SFI1 from humans and yeast (Figure S1) [12,40]. No repeats are present in the highly disordered C-terminal portion of TgSFI1 (Figure S1). The sequence logo shows the motifs derived from this analysis (Figure 1A). Interestingly, all repeats identified in TgSFI1 have a Trp at position 17 (position 1 of the hydrophobic triad) in the sequences defined as centrin-binding sites [12,13]. Compared with position 17, positions 14 and 10 (positions 4 and 8 of the hydrophobic triad) are less strict in terms of residue preference, and allow the presence of Phe, Leu, Trp, Val, or Ile.

Based on this information and the positions of the crucial hydrophobic residues in the triad, seven peptides corresponding to different representative repeats of the TgSFI1 protein were selected. Figure 1B shows the seven peptides (all of comparable size, 18–20 residues) that were chemically synthesized and tested to characterize and compare the binding properties of TgCEN1 to the different potential centrin-binding sites of TgSFI1. TgCEN1 is highly flexible due to the unrelated movements of its two lobes, and to the long N-terminal string (21 residues) exhibiting a high intrinsic disorder [31]. In addition, the N-terminal extension was shown to prompt the formation of heterogeneous Ca^2+^-dependent self-assemblies [19]. Decreasing the self-assembly propensity and the molecular disorder by removing this N-terminal fragment enabled us to significantly improve the quality of the data, especially for analyses requiring high protein concentrations. Thus, for all the experiments in this work, we used a recombinant variant of TgCEN1 that lacks the first 21 N-terminal residues.

### 3.1. Energetics of TgCEN1 Binding to TgSFI1 Repeats

**Figure 2:**
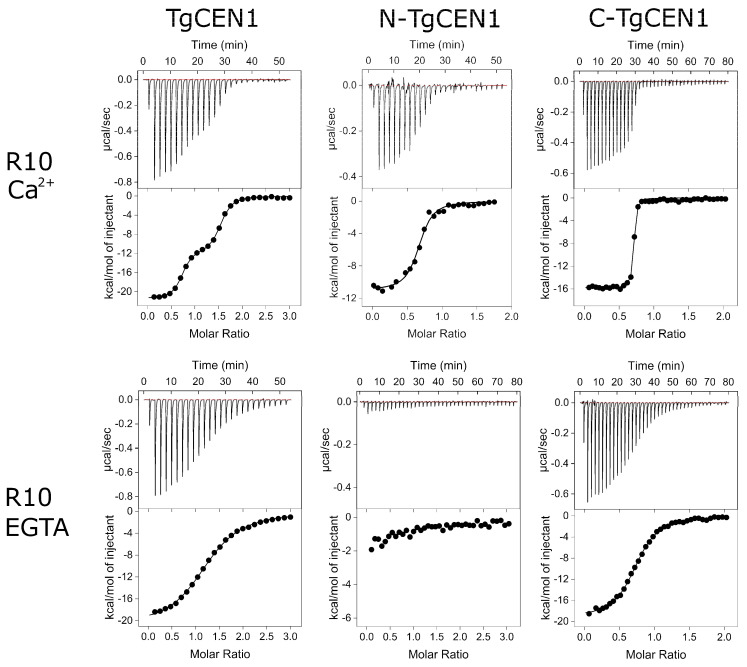
ITC characterization of the interactions of TgCEN1 and its domains with the R10 peptide in the presence of CaCl_2_ or EGTA. Representative thermograms (top panels) and the derived binding isotherms (bottom panels) of titration of R10 into TgCEN1, N-TgCEN1, and C-TgCEN1 in the presence of 5 mM CaCl_2_ or 5 mM EGTA at 25 °C. The ligand dilution blank experiments (peptide titrated into buffer) were subtracted from the binding isotherm obtained in the presence of protein. The first injection of 0.2 μL was made, and then the first data point was removed from data fitting.

The binding properties of the seven repeats were first studied using isothermal titration calorimetry (ITC), which allows the measurement of the affinity constant and thermodynamic parameters (ΔH and ΔS) of molecular interactions. Experimental data for the binding of TgCEN1 and its individual N- and C-lobes for some representative peptides in the presence and absence of Ca^2+^ are shown in Figure 2, whereas the thermodynamic parameters accompanying the binding events are summarized in Table 1. First, we measured the energetics of the interaction between TgCEN1 and the R10 or R16 peptides containing the typical WLL triad. The ITC experiments showed that the binding of R10 to TgCEN1 in the presence of Ca^2+^ is exothermic and is described by a two-step process, such as the one described for the previously reported XPC-TgCEN1 interaction (Figure 2) [19]. The first step, which indicates the binding of the first peptide to TgCEN1, has a dissociation constant (K_d_) ~10^−9^ M, whereas the second step, which corresponds to the binding of a second R10 peptide to Ca^2+^-TgCEN1, has a K_d_ on the order of 10^−6^ M, revealing a much weaker affinity. In contrast, in the presence of the chelating agent EGTA, the curve showed a single site with a K_d_ of ~0.8 µM. To unambiguously assign the different dissociation constants to the individual lobes of the protein, the isolated N-domain (N-TgCEN1) and the C-terminal domain (C-TgCEN1) of TgCEN1 were used. In the presence of Ca^2+^, 1:1 stoichiometry was observed for each lobe, and the C-TgCEN1 had a higher affinity (K_d_ ~27 nM) than the N-TgCEN1 (K_d_ ~1.2 µM), indicating that the sites of high and low affinity in the intact protein correspond to the C- and N-terminal domains, respectively. In the absence of Ca^2+^, no binding was observed for N-TgCEN1, whereas a single interaction was observed for C-TgCEN1 with a K_d_ ~1.8 µM, indicating that the only binding site in TgCEN1 in the presence of EGTA is in the C-lobe. Thus, the two domains of TgCEN1 interact with R10 in different ways; the N-lobe binds R10 in a strictly Ca^2+^-dependent manner and with moderate affinity, whereas the C-lobe binds the peptide with very high affinity and poor Ca^2+^ sensitivity. A similar result was obtained when the interactions of both Ca^2+^-bound and apo-TgCEN1 and its domains with the R16 peptide were analyzed (Table 1), suggesting a similar binding mode of TgCEN1 to the two peptides, which contain the typical WLL triad with very similar thermodynamic behavior and 2:1 stoichiometry.

**Figure 3:**
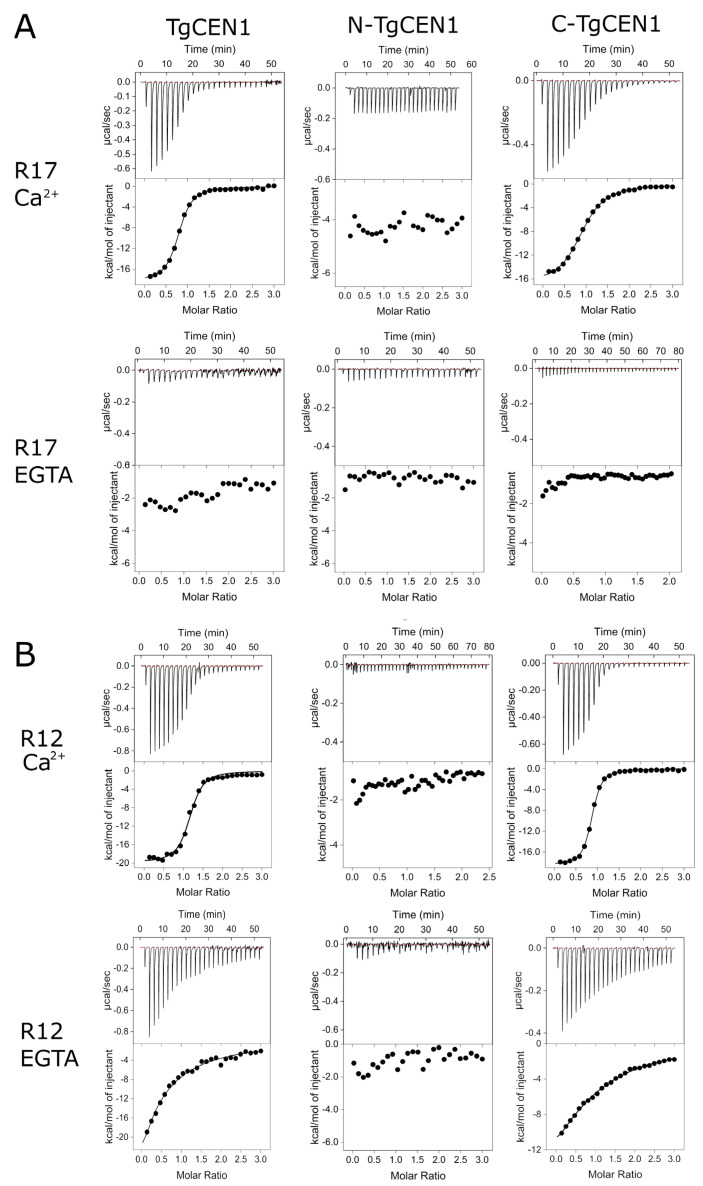
ITC characterization of the interactions of TgCEN1 and its domains with the R17 and R12 peptides in the presence of CaCl_2_ or EGTA. Representative thermograms (top panels) and the derived binding isotherms (bottom panels) of titration of R17 (**A**) and R12 (**B**) into TgCEN1, N-TgCEN1, and C-TgCEN1 in the presence of 5 mM CaCl_2_ or 5 mM EGTA at 25 °C. The ligand dilution blank experiments (peptide titrated into buffer) were subtracted from the binding isotherm obtained in the presence of protein. A first injection of 0.2 μL was made and then the first data point was removed from data fitting.

Interestingly, this binding mode was very different from findings for peptides R17, R24, and R31 (Figure 3). R17 and R24 contain a Phe in the fourth position of the triad, whereas R31 has a Trp in the same position (Figure 1B). Phe and Trp are quite frequent in the fourth position of the TgSFI1 repeats (12/32 and 9/32, respectively) (Figure 1A). All three peptides contain a conserved Leu at position 8. R24 has a Pro residue in the sequence, which is thought to reduce the binding affinity, probably as a result of its rigidifying effect on the peptide conformation [12]. Thermograms for Ca^2+^-bound TgCEN1 in the presence of each of the three peptides (Figure 3A shows R17 as an example) showed a single exothermic event, and data analysis indicated that the integral protein TgCEN1 binds the peptides with a stoichiometry of 1:1 and a K_d_ on the order of 10^−6^ M. Remarkably, no interaction was observed in the absence of the divalent ion, pointing to strict Ca^2+^-dependent binding. Calorimetric titrations using C-TgCEN1 gave very similar affinity values, whereas titration using N-TgCEN1 yielded no binding, suggesting that peptide binding for R17, R24, and R31 mainly involves residues of the C-terminal domain of TgCEN1. 

Peptides R12 and R5 also showed significant binding to TgCEN1 (Figure 3B). In these peptides, Trp1 is conserved, Leu4 is substituted with Phe or Trp, and Leu8 is the least conserved residue with Val or Ile substitutions (in R5 and R12, respectively) (Figure 1B). In the presence of Ca^2+^, the binding isotherms best fit a single-binding site model, with a K_d_ of ~0.5 μM. Interestingly, after removal of Ca^2+^, the affinity of R12 and R5 for TgCEN1 was significantly weaker, but their binding ratio remained unchanged 1:1 (Figure 3B). Similar values were obtained with the C-TgCEN1 construct, clearly identifying the C-lobe as the primary TgCEN1 binding region (Figure 3B). Thus, TgCEN1 forms a 1:1 complex with R5 or R12 via its C-lobe with moderate Ca^2+^-sensitivity.

The collective data from ITC suggest that TgCEN1 has different binding modes in terms of stoichiometry, affinity, and Ca^2+^ dependence with the internal consensus repeats in TgSFI1. Remarkably, all interactions are driven by enthalpic and not entropic effects, suggesting that electrostatic contacts must be important, in addition to the known importance of the interaction between the hydrophobic pockets of centrins with the hydrophobic triad of target sequences. 

### 3.2. Conformational Features of the Interaction

**Figure 4:**
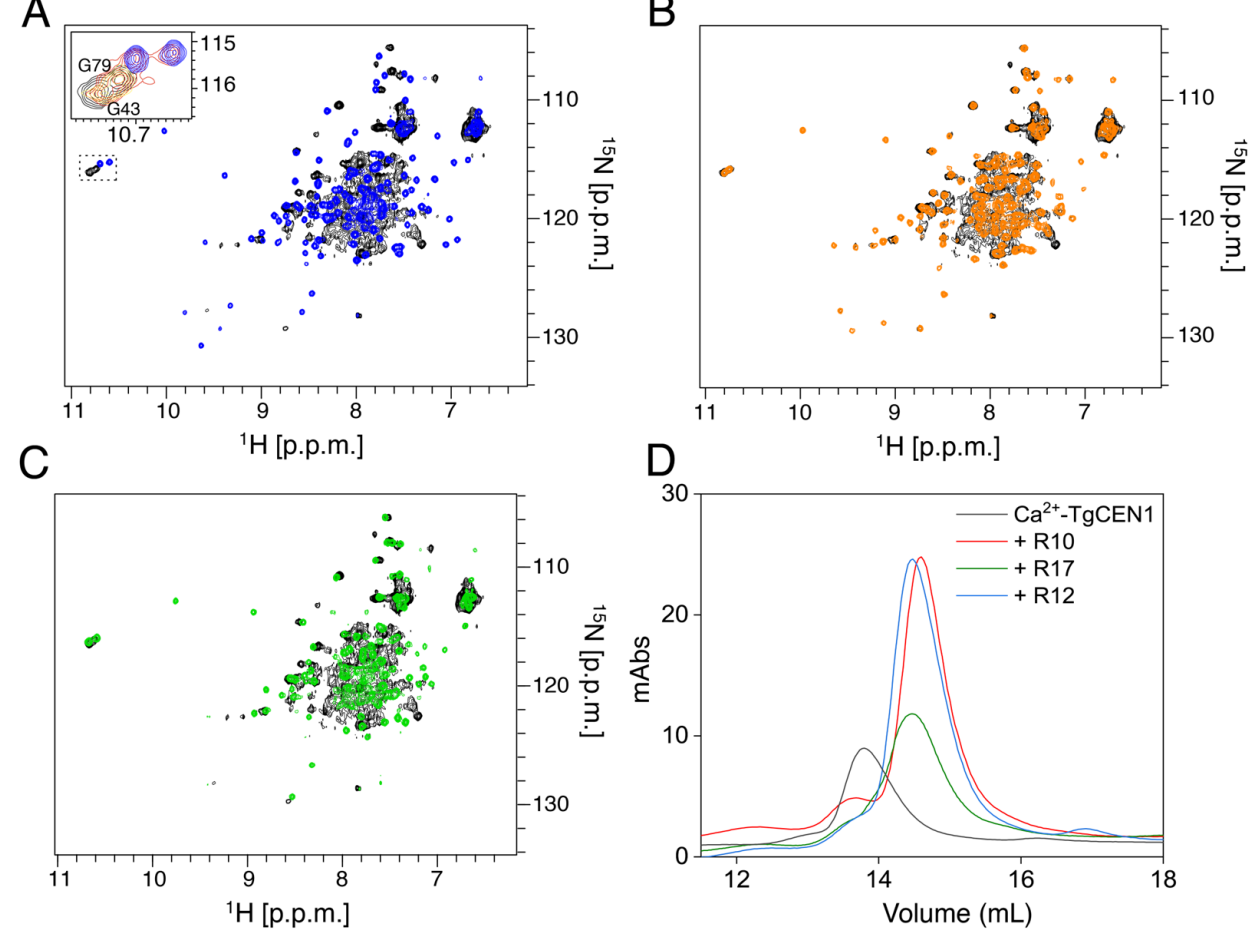
Conformational features of Ca^2+^-TgCEN1-peptide interaction. (**A**) ^1^H-^15^N-HSQC spectra of Ca^2+^-TgCEN1 in the absence (black) and presence (blue) of 2.5 molar excess of R10. The figure inset box contains the chemical shift changes of the two N-terminal glycines at position 6 of the EF-hand binding loop (G43 and G79) during the titration. Selected protein:peptide molar ratios are 0 (black), 0.5 (yellow), 1 (red), and 2.5 (blue). (**B**) ^1^H-^15^N-HSQC spectra of Ca^2+^-TgCEN1 in the absence (black) and presence (orange) of 2.5 molar excess of R17. (**C**) ^1^H-^15^N-HSQC spectra of Ca^2+^-TgCEN1 in the absence (black) and presence (green) of 2.5 molar excess of R12. (**D**) SEC profiles of Ca^2+^-TgCEN1 alone (black line) and in complex with R10 (red line), R17 (green line) or R12 (blue line) peptides. All runs were performed on a Superose 12 10/300 GL column in buffer containing 5 mM CaCl_2_. In all runs, the same concentration of Ca^2+^-TgCEN1 (2 mg/mL) was used and a 1:2 molar ratio of R10 peptide and 1:1 molar ratio of R17 or R12 peptides was added.

Additional support for the different binding modes of TgCEN1 with R10, R17, and R12 peptides was provided by NMR experiments which give information on ligand-induced chemical shift changes [42]. As shown in Figure 4, adding R10 (Figure 4A, blue), R17 (Figure 4B, orange) or R12 (Figure 4C, green) peptides to the ^15^N Ca^2+^-TgCEN1 protein solution (black) induces important changes, resulting in a widely dispersed spectrum with many ^1^H-^15^N resonances that undergo appreciable chemical shift perturbation. The observed spectral changes suggest that the protein experiences important conformational rearrangements upon binding to each peptide and forms a well-structured complex, consistent with the high value of the binding constant measured by ITC.

Interestingly, careful analysis via ^1^H-^15^N-HSQC titration experiments indicated the presence of two R10 binding sites on Ca^2+^-bound TgCEN1 that differ in affinity (Figure 4A). Up to a stoichiometric ratio of 1:1, binding is consistent with slow exchange behavior (typically associated with high binding affinity [43]). The resonances of the free Ca^2+^-TgCEN1 disappear, whereas those for the TgCEN1-R10 complex become apparent. In contrast, the binding of the second peptide is characterized by intermediate exchange on NMR timescale with line broadening and disappearance of signals during the titration, followed by reappearance of signals at a new position. This effect indicates a much weaker affinity for this second interaction [44]. Notably, the resonances of G43 and G79 residues, which correspond to the conserved glycine at position 6 of the first and second N-terminal EF-hands [31], appear to be almost undisturbed up to a molar ratio of 1:0.5 protein:peptide, whereas at a molar ratio 1:1, the two resonances are severely broadened and display a significant intensity loss (inset box Figure 4A). Above this ratio, a change in the chemical shift and sharpening of these resonances was observed. These results indicate that the second binding event involves the G43 and G79 residues, consistent with the N-terminal domain being the low affinity site.

Titration of ^15^N labeled Ca^2+^-TgCEN1 with unlabeled R17 gave spectra describing a 1:1 stoichiometry. Addition of 0.5 equivalents of peptide resulted in the appearance of new resonances in the ^1^H-^15^N-HSQC spectrum of Ca^2+^-TgCEN1, which reached the maximum of intensity at a 1:1 molar ratio (Figure 4B). Only one single set of TgCEN1 resonances was visible when bound to R17, and further additions of the ligand did not produce any significant change (Figure S2). Notably, no changes in G43 and G79 resonances were visible during titration experiments, suggesting that the C-terminal domain of TgCEN1 is sufficient for binding to R17. A similar outcome was seen when the interaction of Ca^2+^-bound TgCEN1 with the R12 peptide was examined through ^1^H-^15^N-HSQC titrations, pointing to a 1:1 stoichiometry (Figure 4C).

We also analyzed the solution properties (Stokes radius, R_s_) of Ca^2+^-TgCEN1 alone and in complex with the R10, R17 or R12 peptides using size exclusion chromatography (SEC) (Figure 4D). Notably, the binding of each peptide to Ca^2+^-TgCEN1 determined a decrease in R_s_ (the protein–peptide complex elutes from SEC later than Ca^2+^-TgCEN1 alone) (Table S1). This implies that TgCEN1 undergoes a conformational change in the presence of each peptide and the final complex has a smaller R_s_ than Ca^2+^-TgCEN1, which could be consistent with a more compact structure. These data are in agreement with the considerable spectral changes of ^15^N TgCEN1, monitored by ^1^H-^15^N-HSQC experiments.

**Figure 5:**
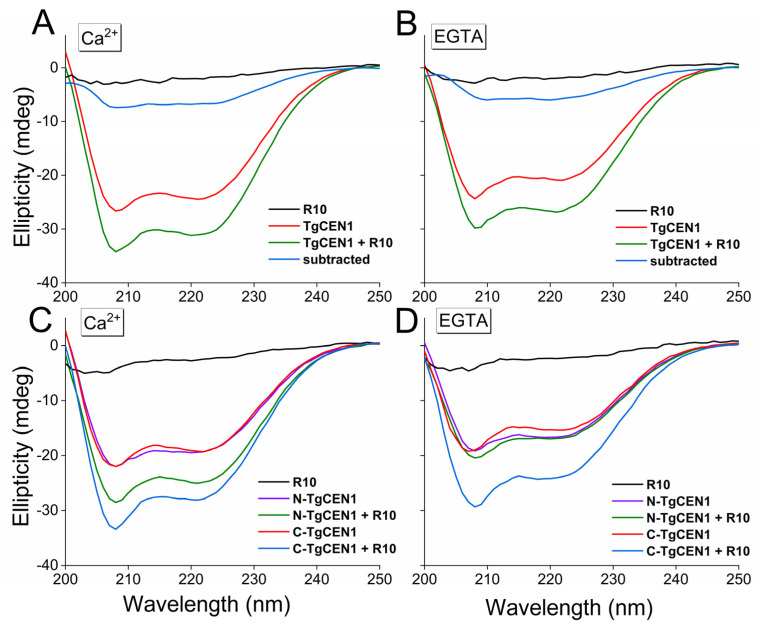
Far-UV CD analysis of R10 binding to intact TgCEN1 and its isolated domains in the presence of CaCl_2_ or EGTA. (**A**,**B**) Far-UV CD spectra of R10 peptide alone (black line), TgCEN1 (red line), and protein–peptide complex (green line) in the presence of (**A**) 5 mM CaCl_2_ or (**B**) EGTA. The CD spectrum resulting from subtraction of the spectrum of protein–peptide complex from that of protein alone was also shown (blue line). (**C**,**D**) Far-UV CD spectra of R10 peptide alone (black line), N-TgCEN1 (violet line), or C-TgCEN1 (red line), N-TgCEN1–peptide complex (green line) or C-TgCEN1–peptide complex (blue line) in the presence of (**C**) 5 mM CaCl_2_ or (**D**) EGTA. In all spectra, the same concentration of TgCEN1 (0.2 mg/mL) was used, and a 1:2 molar ratio of R10 peptide was added. For the single domains, the same concentration of N-TgCEN1 or C-TgCEN1 (0.2 mg/mL) was used, and a 1:1 molar ratio of R10 peptide was added. Increasing the peptide-to-protein ratio was not accompanied by obvious CD changes, in perfect agreement with the ITC-obtained stoichiometry.

The thermodynamic parameters determined by ITC indicate that the binding process for all peptides is driven by an enthalpic component that balances the negative entropy variation. It could be that the increase in the entropy of the solvent is compensated by other events, such as the decrease in entropy of the target peptide, since it assumes a more helical conformation in the final complex. To elucidate this aspect, we collected the far-UV CD spectra of the isolated molecules (TgCEN1 and peptides) and the bimolecular complexes. We chose peptides R10, R17, and R12 as representative peptides for the three different binding modes of TgCEN1 to the repeats of TgSFI1 identified by ITC experiments. All the free peptides have a random coil-type spectrum in solution (Figure 5, Figures S3 and S4, black line). In contrast, TgCEN1 and its isolated lobes are well structured with both Ca^2+^ and EGTA, and show spectral features typical of a protein with mainly α-helical structure (minima at 208 and 222 nm), as previously reported (Figure 5, Figures S3 and S4) [17,19,31].

Addition of R10 peptide to the intact protein and to both the N- and C-lobes resulted in a significant increase in the ellipticity signal in the presence of Ca^2+^ as a result of the peptide contribution (Figure 5A,C). Indeed, this increase in the α-helices content can reasonably be attributed to a random coil-to-helix structural transition of the peptide upon binding to the protein variants (as highlighted by the subtraction spectra, where the spectrum of TgCEN1 is subtracted from that of the protein–peptide complex, Figure 5A, blue line), in accordance with many other centrin-binding targets [8,11,12,19,20]. In the absence of Ca^2+^, addition of R10 peptide to C-TgCEN1, but not to N-TgCEN1, resulted in the same ellipticity changes (Figure 5B,D), confirming the hypothesis that binding of R10 to the N-lobe of TgCEN1 is strictly Ca^2+^ dependent, whereas binding of R10 to the C-lobe is characterized by low Ca^2+^ sensitivity.

Interestingly, in the case of the R17 peptide, the enhancement of the CD signal was observed only with intact TgCEN1 and its C-lobe in the presence of Ca^2+^ (Figure S3), confirming that TgCEN1 can bind this specific repeat in its C-terminus only in the presence of Ca^2+^, with the peptide adopting a helical structure in the final complex. Compared with R17, the addition of R12 peptide results in a similar increase in spectral intensity only for TgCEN1 or C-TgCEN1, regardless of whether Ca^2+^ ions were added or removed, supporting the formation of a complex with moderate Ca^2+^ sensitivity thorough the C-lobe (Figure S4).

In order to obtain further information on the peptide conformational rearrangements upon binding to Ca^2+^-TgCEN1, isotope filtered NMR experiments were performed on a ^15^N-labeled Ca^2+^-TgCEN1-unlabeled peptide complex [45]. Generally, the presence of compact states in unstructured peptides and intrinsically disordered molecules is too poor to give suitable ^1^H-^1^H contacts [46]. However, as shown in Figure S5, several intramolecular NOESY cross peaks appeared in the double filtered NOESY experiment upon complex formation, in agreement with the peptide undergoing conformational changes toward a more ordered structure. These results comply with the far-UV CD spectra of the single peptides recorded in the presence of varying amounts of 2,2,2-Trifluoroethanol (TFE) (Figure S6). TFE is well known to induce α-helical structure in peptides that possess the propensity to be helical. As shown in Figure S6 in aqueous solution, all the peptides are largely unstructured, with a negative peak at ~198 nm, while when the TFE concentration is increased to 20%, they start to adopt an α-helical structure, with two peaks at 208 and 222 nm. The helical content increases further, reaching 80% TFE. These data provide evidence that the analyzed TgSFI1 peptides have a high propensity to undergo structural change upon binding of a ligand, supporting the ability of TgCEN1 to induce α-helical structure in its binding targets.

### 3.3. Trp Residue of TgSFI1 Repeats Is Embedded in TgCEN1 Hydrophobic Pocket

To further support the different binding modes of TgCEN1 to sequence repeats of TgSFI1, the interaction between TgCEN1 and the different peptides was then studied by Trp fluorescence spectroscopy. It is known that the emission of the Trp indole moiety is sensitive to the different features of the microenvironment. TgCEN1 does not have Trp residue, whereas all TgSFI1 peptides have at least one Trp in the hydrophobic triad, making them sensitive probes to study the interaction between TgCEN1 and its binding partners.

**Figure 6:**
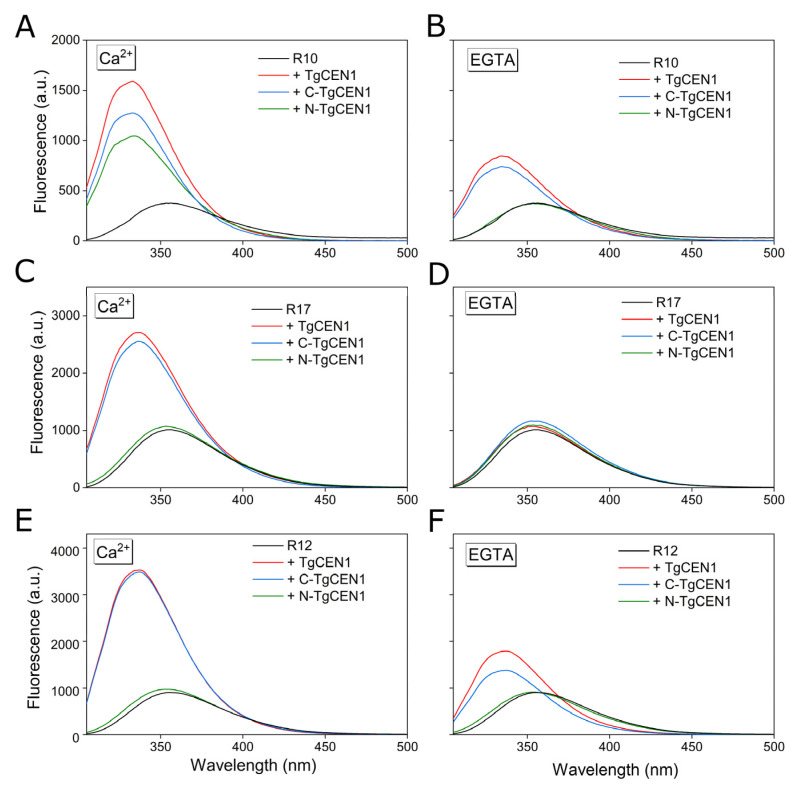
Binding of TgCEN1 and its domains to the different peptides in the presence of CaCl_2_ or EGTA measured by Trp fluorescence. Trp fluorescence emission spectra of peptide alone (black line), and upon addition of intact TgCEN1 (red line), C-TgCEN1 (blue line), or N-TgCEN1 (green line) in the presence of CaCl_2_ (**A**,**C**,**E**) or EGTA (**B**,**D**,**F**).

We analyzed the emission properties of R10, R17, and R12 after addition of TgCEN1 in the presence and absence of Ca^2+^. As shown in Figure 6, the spectra of the three peptides alone (black line) exhibited a fluorescence maximum between 354–356 nm, indicating the exposure of the Trp to the polar solvent. Addition of TgCEN1 (red line) to all peptides in the presence of Ca^2+^ (Figure 6A,C,E) resulted in a considerable increase in fluorescence emission and in a shift of the maximum emission to shorter wavelengths (blue shift), both typical of Trp being embedded in a more hydrophobic environment. This behavior clearly indicates that Trp appears to be shielded from solvent quenching when bound to Ca^2+^-TgCEN1, confirming the crucial role of Trp residue of each peptide in the formation of the complex.

Similar effects were observed after adding the C-domain (C-TgCEN1, blue line) to all the three peptides (Figure 6A,C,E), and the N-domain (N-TgCEN1, green line) to R10 peptide (Figure 6A). In the absence of Ca^2+^, both the blue-shift and fluorescence enhancement effects were smaller, but still observed upon addition of TgCEN1 (red line) or C-TgCEN1 (blue line) to R10 and R12, but not with N-TgCEN1 (green line) (Figure 6B,F). In contrast, no changes were observed upon addition of the intact protein or its single domains to R17 peptide (Figure 6D).

## 4. Discussion

Investigating the molecular mechanisms that govern centrin–target interactions is crucial to understanding the ability of centrin to recognize and bind multiple biological target proteins. Similar to CaM, centrins are supposed to possess an extremely versatile structure. They can position their domains, tune their Ca^2+^ sensitivity in the presence of diverse partners, and compete or interact dynamically with other proteins.

Herein, our goal was to characterize the molecular interactions that lead to the formation of the TgCEN1-TgSFI1 complex, and the conformational changes associated with the interaction compared to the single components. We used a peptide model approach to simulate a target protein that has been employed in many studies of other Ca^2+^ binding proteins and their regulatory interactions [8,12,18,20,23,47,48], and has demonstrated, in many cases, the ability to uncover crucial determinants of the interaction.

**Figure 7:**
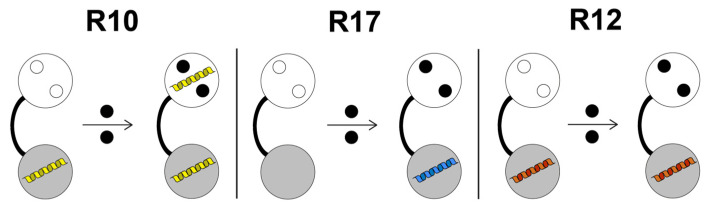
Schematic illustration of the binding modes of TgCEN1 with different repeats from TgSFI1. TgCEN1 is shown as a dumbbell, indicating its two-domain structure (N- and C-lobes in white and gray, respectively); open circles denote unoccupied Ca^2+^-binding sites; filled black circles are Ca^2+^ ions. Yellow, blue, and red α-helices denote the representative R10, R17, and R12 target peptides, respectively. TgCEN1 exhibits two binding sites for R10 with distinct affinities: the C-lobe binds the peptide with very high affinity and poor Ca^2+^ sensitivity, while the N-lobe binds R10 with moderate affinity only in the presence of Ca^2+^. TgCEN1 binds R17 with moderate affinity only via the C-lobe in a strictly Ca^2+^-dependent manner. TgCEN1 interacts with R12 through the C-terminal domain with moderate Ca^2+^ sensitivity.

We analyzed the parameters involved in TgCEN1 binding to seven repeats of TgSFI1 that were selected among the 32-putative centrin-binding sites identified in the TgSFI1 sequence [29]. Overall, our data indicate that most of the repeats in the intact protein represent a binding site for TgCEN1. However, the different repeats bind centrin with various affinities, different stoichiometry, and levels of Ca^2+^ dependence, suggesting that each TgSFI1 repeat has evolved to use different properties of the versatile TgCEN1 architecture (Figure 7).

In agreement with several other centrins in which the C-lobe is primarily involved in the complex formation [11,12,14,21,22,49,50], our data clearly identify that the C-terminal domain is the primary TgCEN1 target binding region. We found that TgCEN1 binds many repeats with 1:1 stoichiometry through its C-terminal domain. However, analysis of the interaction between TgCEN1 and either R10 or R16 peptides, which contain a typical W^1^L^4^L^8^ triad, indicates the presence of a second potential binding site in the N-lobe of TgCEN1, supporting a specific mechanism for binding in these repeats in which the C-lobe of TgCEN1 is constitutively bound, with high affinity, to TgSFI1, independent of Ca^2+^. The N-lobe constitutes a sort of Ca^2+^ sensor and only binds the peptide in the presence of Ca^2+^ with lower affinity (Figure 7).

In contrast with the notion that the interaction of SFI1 with centrins is only moderately dependent on Ca^2+^, we found that the interaction between TgCEN1 and some peptides (e.g., R17, R24, and R31 repeats) is strictly Ca^2+^ dependent, similar to the Ca^2+^ regulation of many CaM protein targets (Figure 7). The Ca^2+^ dependence observed in these protein–peptide interactions, along with the Ca^2+^ dependence between the N-lobe of TgCEN1 and R10 (or R16) peptide, suggests that, in addition to the constitutive binding between some TgCEN1 molecules and specific repeats of SFI1, which is consistent with the colocalization of TgCEN1 and TgSFI1 [29], a Ca^2+^ stimulus can determine new interactions of TgCEN1 with other repeat sequences of TgSFI1. This would permit TgCEN1 to work as a Ca^2+^-dependent “adaptor” protein, with a dynamic role in organizing large protein assemblies. Interestingly, the crystal structure of CDC31 bound with some repeats of ScSFI1 revealed that both the C- and N-lobe of the centrin can bind ScSFI1 [27]. The observed variation in Ca^2+^ sensitivity, as well as in Ca^2+^ affinity, is most likely the consequence of the sequence diversity between the SFI1 repeats. Various binding behaviors have also been observed for distinct human SFI1 repeats [12]. Notably, the crystals of some SFI1–centrin complexes have been obtained only with Ca^2+^ [27] and it was recently suggested that Ca^2+^ stabilizes the SFI1–centrin complexes by increasing their thermal stability [40]. 

One limitation of the present study is that a peptide can freely bind TgCEN1 in conformations that are less constrained compared to the intact protein. The space between adjacent TgSFI1 repeats is compatible with centrin–centrin interactions that could affect the TgSFI1-TgCEN1 interaction; thus, the thermodynamic parameters found with the TgSFI1-derived peptide may not be reflective of those *in vivo*. Furthermore, the interplay between the three different centrins identified in *T. gondii* may add further complexity. However, even if it remains elusive as to how TgCEN1 exerts its regulatory role *in vivo*, the various binding behaviors observed with our distinct TgSFI1 repeats clearly indicate an inherent flexibility and plasticity of TgCEN1 in binding its biological targets. This flexibility seems to be dictated by the multiple target binding motifs, their distribution, low sequence complexity, and amino acid composition, with the hydrophobic triad working as major determinant of the interaction. It is well established that the bulky hydrophobic residues 1 and 4 of the triad are critical for centrin binding, and serve as key anchor points. We found that the Trp residue in position 1 is absolutely conserved in TgSFI1, in good agreement with the crystal [5,27,49,51] and NMR [22,52,53,54] structures of centrins complexed with peptide target sequences, showing that Trp1 is buried within the binding cavity of centrin. Such a strict conservation is not found at position 4, where Phe (12/32), Leu (11/32) or Trp (9/32) residues are found. Similar to SFI1 homologues from other organisms, a Leu residue is mainly found at position 8 (26/32) in TgSFI1 repeats. Interestingly, the replacement of Trp1 by Ala was shown to significantly decrease the affinity of the binding, while substitution of Leu at position 4 with Ala has a less dramatic effect, and substitution of the eighth residue of the triad does not influence the affinity constants [8,51], supporting its minor role in the binding. In addition to the hydrophobic interactions, the comparison of the amino acid sequences of the TgSFI1 repeats reveals the presence of a series of basic amino acids interspersed with hydrophobic residues that can undergo electrostatic interactions with the negatively charged surface of TgCEN1, likely having different effects on binding with TgCEN1. Accordingly, our ITC data indicate that all interactions are driven by enthalpic and not entropic effects, suggesting that, although charge effects appear to be of secondary importance to nonpolar effects, they contribute to the stability of the complex, and the different TgCEN1 modes of binding might be determined by an interplay of specific hydrophobic and electrostatic interactions.

The structural plasticity of TgCEN1 is also a result of the target sites being intrinsically disordered and folding upon the binding of TgCEN1. Our CD and NOESY spectra clearly support the induction of α-helical structure in the centrin-binding sequences of TgSFI1, reflecting the importance of this step in the formation of the complex. Moreover, TgCEN1 undergoes a disorder-to-order transition, as shown by NMR spectra.

The distinct binding modes observed in TgCEN1 appear to be crucial to allow its dynamic association with TgSFI1, supporting the formation of a complex in which multiple centrin molecules bind continuously along the SFI1 backbone and adopt different wrap-around conformations to produce a filament-like structure. In this scenario, the previously reported Ca^2+^-dependent ability of TgCEN1 to self-assemble in a N-to-C mode [19] could suggest an additional level of sophistication in the TgCEN1-TgSFI1 complex formation and in the regulatory role of TgCEN1 in centrosome architecture and centriole duplication.

Undoubtedly, the binding of TgCEN1 to the TgSFI1 repeats remains an intriguing structural problem, and further detailed structural analyses of the TgCEN1-TgSFI1 complexes in the presence and absence of Ca^2+^ will be required to better understand the dynamic behavior of the centrosome.

## Figures and Tables

**Table 1:** Summary of the thermodynamic parameters of the interaction between TgCEN1 and its N-terminal and C-terminal domains with the peptides derived from TgSFI1. n—stoichiometry of binding; K_d_—dissociation constant; T—298 K.

	Buffer	n	K_d_ (µM)	ΔH (kcal mol^−1^)	TΔS (kcal mol^−1^)
**TgCEN1 + R10**	CaCl_2_	n_1_ = 0.7 ± 0.1	0.014 ± 0.002	−19.1 ± 0.4	−6.3 ± 0.4
		n_2_ = 0.9 ± 0.1	2.5 ± 0.3	−12.1 ± 0.3	−4.4 ± 0.3
	EGTA	0.8 ± 0.1	0.8 ± 0.1	−24.7 ± 0.4	−16.4 ± 0.4
**N-TgCEN1 + R10**	CaCl_2_	0.7 ± 0.1	1.2 ± 0.4	−12.6 ± 0.9	−4.4 ± 1.0
	EGTA	-	-	-	-
**C-TgCEN1 + R10**	CaCl_2_	0.7 ± 0.1	0.027 ± 0.005	−15.8 ± 0.1	−5.5 ± 0.5
	EGTA	0.7 ± 0.1	1.8 ± 0.1	−19.3 ± 0.1	−11.5 ± 0.7
**TgCEN1 + R16**	CaCl_2_	n_1_ = 0.7 ± 0.1	0.009 ± 0.001	−20.9 ± 2.5	−9.9 ± 2.4
		n_2_ = 0.8 ± 0.1	0.5 ± 0.1	−11.8 ± 2.7	−3.3 ± 1.7
	EGTA	0.9 ± 0.2	3.4 ± 0.3	−26.2 ± 3.4	−18.7 ± 3.3
**N-TgCEN1 + R16**	CaCl_2_	0.7 ± 0.1	0.4 ± 0.1	−11.6 ± 0.6	−2.8 ± 0.7
	EGTA	-	-	-	-
**C-TgCEN1 + R16**	CaCl_2_	0.7 ± 0.1	0.006 ± 0.001	−18.8 ± 1.5	−8.9 ± 1.6
	EGTA	0.8 ± 0.1	9.1 ± 0.8	−22.4 ± 0.6	−15.7 ± 2.2
					
**TgCEN1 + R17**	CaCl_2_	0.7 ± 0.1	0.8 ± 0.2	−18.1 ± 0.5	−9.7 ± 0.7
	EGTA	-	-	-	-
**N-TgCEN1 + R17**	CaCl_2_	-	-	-	-
	EGTA	-	-	-	-
**C-TgCEN1 + R17**	CaCl_2_	0.9 ± 0.1	2.7 ± 0.1	−16.9 ± 0.2	−9.3 ± 0.3
	EGTA	-	-	-	-
**TgCEN1 + R24**	CaCl_2_	0.9 ± 0.1	3.4 ± 0.6	−8.9 ± 0.9	−2.1 ± 0.7
	EGTA	-	-	-	-
**N-TgCEN1 + R24**	CaCl_2_	-	-	-	-
	EGTA	-	-	-	-
**C-TgCEN1 + R24**	CaCl_2_	0.8 ± 0.1	2.6 ± 0.3	−8.6 ± 0.2	−0.9 ± 0.3
	EGTA	-	-	-	-
**TgCEN1 + R31**	CaCl_2_	0.8 ± 0.1	2.9 ± 0.1	−24.2 ± 1.4	−16.6 ± 1.5
	EGTA	-	-	-	-
**N-TgCEN1 + R31**	CaCl_2_	-	-	-	-
	EGTA	-	-	-	-
**C-TgCEN1 + R31**	CaCl_2_	0.8 ± 0.1	1.9 ± 0.1	−22.2 ± 0.3	−14.4 ± 0.3
	EGTA	-	-	-	-
					
**TgCEN1 + R5**	CaCl_2_	0.7 ± 0.1	0.5 ± 0.1	−26.4 ± 2.4	−22.8 ± 1.8
	EGTA	0.6 ± 0.1	36.9 ± 6.6	−24.2 ± 2.1	−10.6 ± 2.3
**N-TgCEN1 + R5**	CaCl_2_	-	-	-	-
	EGTA	-	-	-	-
**C-TgCEN1 + R5**	CaCl_2_	0.7 ±0.1	0.9 ± 0.1	−23.3 ± 0.1	−15.1 ± 0.3
	EGTA	0.9 ± 0.3	47.8 ± 4.9	−10.2 ± 3.1	−14.4 ± 3.2
**TgCEN1 + R12**	CaCl_2_	0.9 ± 0.1	0.5 ± 0.1	−21.5 ± 1.3	−12.7 ± 1.2
	EGTA	1.1 ± 0.1	29.8 ± 3.4	−20.4 ± 2.4	−14.2 ± 2.5
**N-TgCEN1 + R12**	CaCl_2_	-	-	-	-
	EGTA	-	-	-	-
**C-TgCEN1 + R12**	CaCl_2_	0.8 ± 0.1	0.4 ± 0.1	−18.5 ± 0.1	−9.8 ± 0.5
	EGTA	1.2 ± 0.5	69.9 ± 7.0	−13.3 ± 1.9	−7.6 ± 2.9

## Data Availability

Data are available upon request from the corresponding author.

## Supplementary Materials

The following supporting information can be downloaded at: https://www.mdpi.com/article/10.3390/biom12081115/s1, Table S1: Determination of Stokes radius (R_s_) of Ca^2+^-TgCEN1 alone and in the presence of R10, R17 and R12 peptides by SEC. Figure S1: Potential centrin-binding repeats in TgSFI1; Figure S2: NMR intensity analysis of the binding of R17 to ^15^N-TgCEN1. Figure S3: Far-UV CD analysis of R17 binding to intact TgCEN1 and its isolated domains in the presence of CaCl_2_ or EGTA. Figure S4: Far-UV CD analysis of R12 binding to intact TgCEN1 and its isolated domains in the presence of CaCl_2_ or EGTA. Figure S5: 2D ^15^N, ^13^C filtered NOESY spectra of ^15^N-labeled Ca^2+^-TgCEN1 (orange) and of unlabeled R12 peptide bound to uniformly ^15^N-labeled Ca^2+^-TgCEN1 (black). Figure S6: Far-UV CD spectra of 20 μM R10 (A), R17 (B) and R12 (C) peptides in various water/TFE mixtures at 25 °C.
