# Supplementary material for: Conformational Plasticity of Centrin 1 from Toxoplasma gondii in Binding to the Centrosomal Protein SFI1
Source: Biomolecules. 2022 Aug 13;12(8):1115. doi: 10.3390/biom12081115 (PMC9406199; doi:10.3390/biom12081115)
Supplement: Supplementary file 1 [file biomolecules-12-01115-s001.zip › biomolecules-1826901-supplementary.pdf]

Table S1. Determination of Stokes radius (R_s) of Ca^{2+}-TgCEN1 alone and in the presence of R10, R17 and R12 peptides by SEC. The mean values from triplicate experiments and SEM are presented.

	**R_s (nm)**
Ca^{2+}-TgCEN1	2.53 ± 0.02
Ca^{2+}-TgCEN1 + R10	2.17 ± 0.05
Ca^{2+}-TgCEN1 + R17	2.18 ± 0.01
Ca^{2+}-TgCEN1 + R12	2.19 ± 0.02

MLAPTDWTDAEILAAAAQAAREEFFGAQRARVGDTGAQDEARGGGVPFLLVRKQLVRLLDEMGVGLQSTRGLQVY
RHLLQHVVQATVGECGHDMILFSEESRALFMPPVNGAGGTAASRLANANSWSLASSPSVVSSGPAPVLVSASPGP
SGLSDRSFNETTPRVSASSSSAPSSSSSSSPVSFSPSSGPVSSSSTFPPSSSSACPSSSSSSSSSSFSSCSSCSS
SPLASATARVQAFAARRKAGEDHRL**L**LDA**L**RE**W**RRWAAREARLRSLFWTLSSRRRLRA**L**RRS**L**HA**W**RANFIEALS
RREDSNEEAADGQRTAAVCRRSLLAWSAGVETQSAEARGQVARAAPAEEGCQRYEA**L**QTS**F**RS**W**REATVAQREAE
RQRR**L**LSA**F**KA**W**KESHLSRRVRKQVLEHRAFAVQKSLQDRHMRGVWRYWRAAAEARSRAGAAARETLERTRFRRV
FNALLAETRDRQTRRGAAVAAAEATVRERR**V**RRA**F**RA**W**RKVSILRRPPFAAGHSQFSSPQIVCLLKAVSALRREG
ASEEPGHARSLGEALLDAATARQRRERAQRRGDRDGEETELGRDAQVDLEDAALLTLIPLHGIDLHLPKLSTAAP
LMAQALRQHY**L**RHS**F**LQ**W**RAEALRQRQFRQAARVSR**L**ARV**F**RA**W**REETQKAKGEAAASSAVYAAQKTRI**L**REA**W**R
S**W**RMRFRRYVLFRTQCRSLQDLRLLHLKSLAFRGWCTFHRERQREAQSVSAVKDRAAAALQRAAWSRWQEAWHLR
RRRRAMAEAAALLREKTLLAKGLRGLKRQRASAASLRDAAAALRQER**L**GLA**L**AV**W**WRWAARRGAFQELCELQRAK
RARRN**L**RRL**L**LA**W**RSLAKRMETRRDAVWRLAAVVDRIR**V**RRG**F**CG**W**NALRMQERKREEAASRLQRTLRDAR**I**RAA
WAA**W**RSAQSLACKEAELRKESEDSLKRRVFKQWQAAAAEHATLQLHLRGQLLCWR**L**SRL**L**HR**W**RRLATLFRLGLL
QKSRSRLYVLRRCQEAWLARLAERSEERRKEARAESLCLTLRLRAAQRCLLAWQETAVAVAARRRKGDTFRAQLH
RAR**L**DAA**W**TT**W**RRGVEELHRRRRLLLGVEKIREKIL**V**AQS**L**TR**W**KCAAALLRKEALATQARRQAL**L**RRA**L**QA**W**TL
WRRRRTEKKDEAERVRACLNEEGSAKRAGERQRKKETLAAWHALTEKRFEERRAFSRVEAIGDRLS**L**RRG**F**VT**W**R
LWALRLAGLSQATVRLDVWRRRCLLRISYEALSQHRDRVKEMELASLAHLSQLRAQT**L**RRS**F**CS**W**RTQFAALRRE
AEVLARRRSAAARAEAHAALACKRRSFLAWLKALAAKLALKKRLFVLLKERREALEARTLRAWRRLAKKKGDLRE
SLEMFSDSRRSQCLRRVFAAVFATVSRRRLVRVRFLFALGLLAPSPDFSVSSAESSHPHSRSLLLPLSPLGPAEE
ASLRLLWGPALPAPLLAPRLIPRLPGPSRAFESAKECPSVLASLP**L**VPR**L**IV**W**SAQPHMLHLLLLQPAIAESLAR
QAGQQPQRSAAFFEPLPVSRARLALFSDADAEARTRVWGLPAASLGGEETPGGDKGNGALPLAALASARSRPPSV
WSCLTPSTCTPHTSLSVPLSGFEAPPPRPGRTFFRTETGDTLGSARSQFRGRSASPLHSDGPSGREAGSRRHRSA
TKERDREDREEIDELLLADKKSSKSLERLLRCFSAKQGPEGPLKENAELWGEQAVLADSVRRMRPQNDLQALLLA
QVNLSRRPSVQRPAPLFLPSAMGPDAAVSDALDASQDSVESDGSAGGLSVHSEETPLPSKNWREEIPVDLLATAW
QFARL**L**QAA**L**RA**W**LLQAQRHKAQRLRETGLVAGFRIQSRSRL**L**AAC**W**GL**W**RSTKSSLEARTRTKAQRLAWGIQRR
VLQAWFLCATVDASAVLRAEAFAETRRSAKTHAMLEAWRGVATKRRQEREQVEAWRQHRARERKQELFALWTHLA
SVNRAGAQLYVHRRAAT**L**NKV**F**AT**W**RTSTLRSLALDKLQRIVSRPGLFLGFSLLRIWSLTRACSLRLAAQCLDSW
RQHTESRKRLAGLLVYVQTVHKVSR**L**QQH**F**SA**W**LLVQQRRRRLLLLHRTAEAAAFLLP**L**HRI**F**SR**W**RASALDRQA
QRDSLLGRFLAQRQRPFLALDPEESAEAKDAGRLGTTPSCAYWARWR**I**EET**W**KV**W**RRRFRLRTLLRRRATRCART
ALSALRQAVERRRALEAFERKRRQRLLQRATVAWQVYVQRRLLKQQRTAHAIERYNTHTLRQAFSKCRWLLQRLQ
WERGVIEVSQAKANRRVCRMTLQALQAYAEARVAERERLDGCRAARSSHL**L**RMH**F**EK**W**SFLLRATHHAASLSRKT
SFLLWTRAFRRSRGAALLVAVQRRVEERQTRRGLAALQAESEAFEQIKAFGAETPWLRDAIEEKVSSARASMTFA
LSSLRLFAAHRVSRRRQARL**L**AGA**L**AS**W**ARLQETDGPRRR**L**RPY**L**RH**W**RGALQYRLHLQAAEEALRRHVEFRR**L**R
EV**W**GA**W**RHLRREEARGRELLALAEETKKVWALEKGILLLHEHR**L**QRE**W**MT**W**CTYRAELFVTDTRHEKARLCVRAW
HSWVAKRRELRRRGLEAQEASRLFVLSNAFSALRCAYTRRTALRAAGACVVEKQKRSS**L**RTA**W**RG**W**RKVLVDLLA
LKPRLEALLARQAFQR**V**CLA**W**WG**W**RMWHQRRKERRQLLGPLSEKARVVRDVQLAAQCFRGWAELAETRATFWRQR
EGLVEKLVARRLTKETFRAWRLCAREIHFVRSTKAAAANAAALAAAGHGMREMHLPAQAPADRRGRPGPSRETTM
SPASSLFSSASVSPTASRRNRRRGGRSGRPRGRDLLSSSLSLSDSQPLERLSQASDRRGGESQTDGDSGDMSETV
RRRPGCQRPSEAAPGSPPGGRSKRVHAAAGRRRQGAREILEEREISRLARSAVAWLPRAAERSDEAEERALRRRS
ASAVFGSRGRDGESGLGEMRSTTGPAGGHRAWESEASGEDTEGEEGPRNLQGQEAEASTSGKKARGTCGLAVSFS
LGSPLSSDRPSSPLSVAAHEPPPPPGKTRLHRVSSSLSSPRPRGAHAKETPAGPPSSPSSASSASLRQGPRQLRL
QDLLSDSSSSLASAQPAPVFAPQENRGKSKSDSVESPSPLTVATSLLEALSHSHSSALAEAARSLLLQSGARNPD
TKPAASSRDLSESDRSKSFSSADSEMDRTATEDSEDDRGPQGGRRGVRRRRSSPVRRGDRSRRVRREEEEETRRR
STRRRRAAESEQLGEEEDVEEVEEEEVEEEEVEEEEGVSGVEVSLVEKRAEERIRVFPRAIREAPPSAFALPSSL
SSLFLTSLQGAQGSRSAETQAREEPRVGRVLALAHSLAGSASRFERGGDEDRVEDEGQSVESARLSTASLDAGER
DRVPRKGGKRDHPFFFSLMSSTPSLRDQASGGQSSCSSSPASPSSESSVCVGGPRRRAVGAAKEPLGTRDEAKEL
RANCDLAEEPASLAGGAAAHRPDPQGAALTEKLRRIVALAVANRLQSPAAASEEDTEENEGVEKEGADEEEDVVH
SETGGEAENRAERGRLSLISSTLSTTLLLSELAAPQEDEGVPLGSRSAASASHEETGESREEQETTGCERDSPGV
AHRLLLHPATFSLARGSLPPISEGATGSRSSPSPDRLAASTQSEAAAEPTETGDSEAELRKLLARLERVEELHRA
RDFIGAEADDSGALPVANMQSSVDLRSACEAPQDTERQRRRPERMQSPSPPASPSVAPARRLESEAGANRGVPEL
SVSPVAGAQEFVFEGNPDAAETGNEVAEVQRQERRRAAEAEAAVAQNAEDERTEREPRDEEAASGGQREEEEWLQ
DIVAVSRRKAAEARARDQRDESERDARKDTESIAGSEELLEEAWKEARAETRNALLEIHREMKGVALYDGDARDA
SRT

Figure S1. Potential centrin-binding repeats in TgSFI1. The potential centrin-binding sites are highlighted in orange. The hydrophobic triad is in bold.

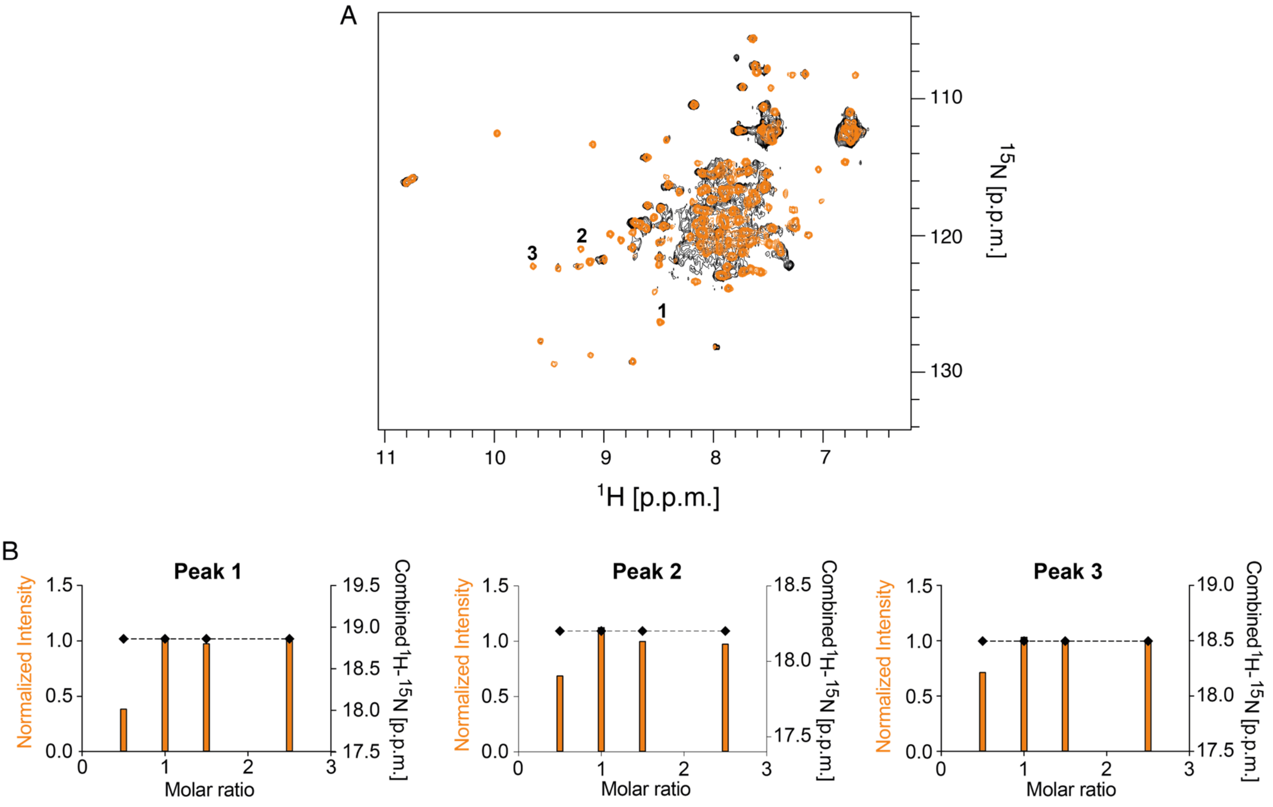

Figure S2. NMR intensity analysis of the binding of R17 to ^{15}N-TgCEN1. (A) ^{1}H-^{15}N-HSQC spectra of TgCEN1 in the absence (black) and presence (orange) of 2.5 molar excess of R17. Selected peaks for the intensity analysis are indicated as 1, 2 and 3. (B) Peak intensity and combined ^{1}H-^{15}N resonance frequency modulation of selected TgCEN1 peaks in function of increasing R17: ^{15}N-TgCEN1 molar ratios. The intensity was normalized by dividing the peak intensity at each titration point by the corresponding peak intensity at 1.5 molar excess of R17 peptide. While the peak intensity increases during the titration, reaching its maximum between 1:1 and 1:1.5 molar ratios, the resonance frequencies remain unperturbed.

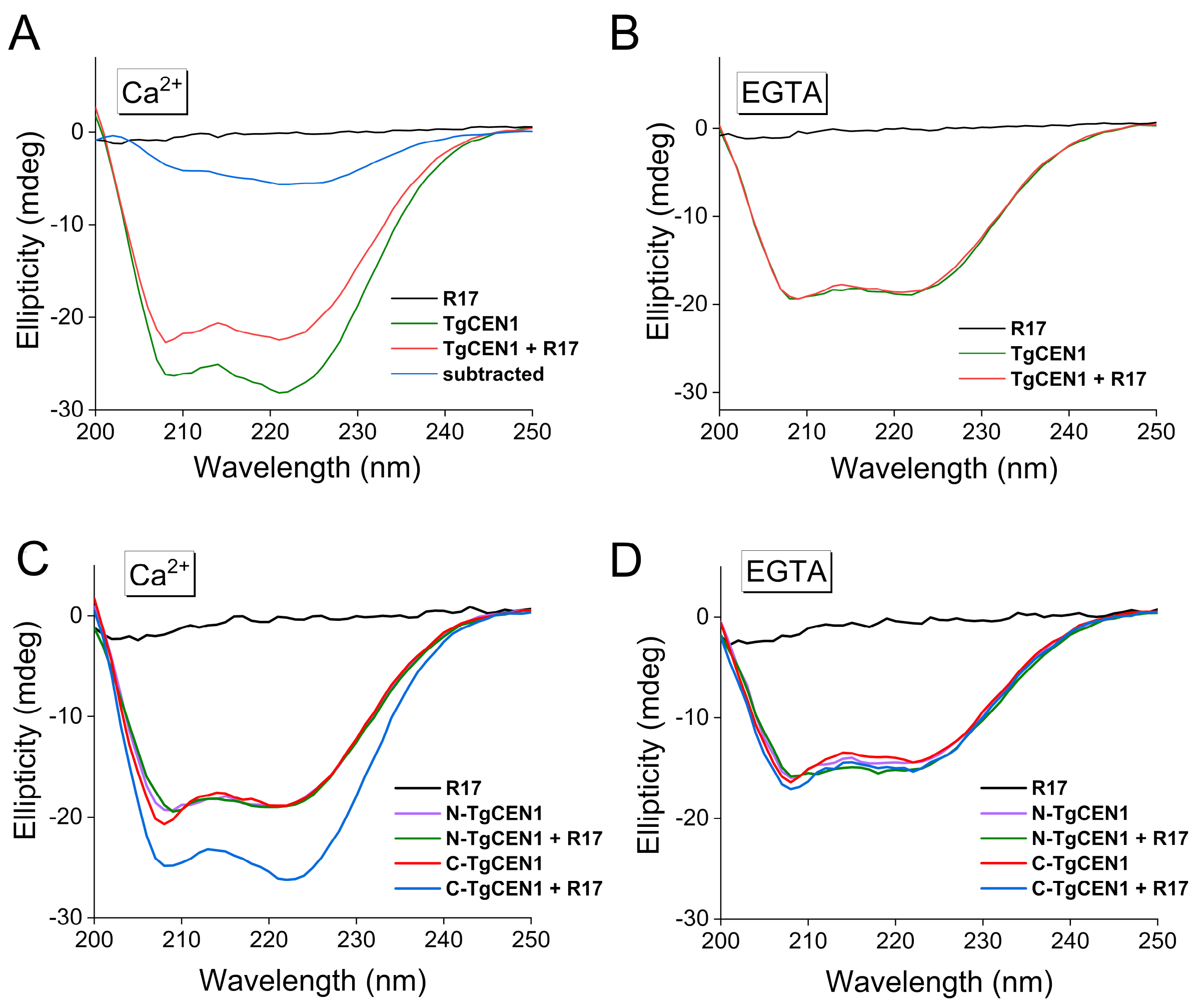

Figure S3. Far-UV CD analysis of R17 binding to intact TgCEN1 and its isolated domains in the presence of $CaCl_2$ or EGTA. (A-B) Far-UV CD spectra of R17 peptide alone (black line), TgCEN1 (red line), and protein-peptide complex (green line) in the presence of (**A**) 5 mM $CaCl_2$ or (**B**) EGTA. The CD spectrum resulting from subtraction of the spectrum of protein-peptide complex from that of protein alone is also shown (blue line). **(C-D)** Far-UV CD spectra of R17 peptide alone (black line), N-TgCEN1 (violet line) or C-TgCEN1 (red line), N-TgCEN1-R17 complex (green line) or C-TgCEN1-R17 complex (blue line) in the presence of (**C**) 5 mM $CaCl_2$ or (**D**) EGTA. In all spectra the same concentration of protein (0.2 mg/mL) was used and a 1:1 molar ratio of R17 peptide was added. Increasing the peptide-to-protein ratio was not accompanied by obvious CD changes, in perfect agreement with the ITC obtained stoichiometry.

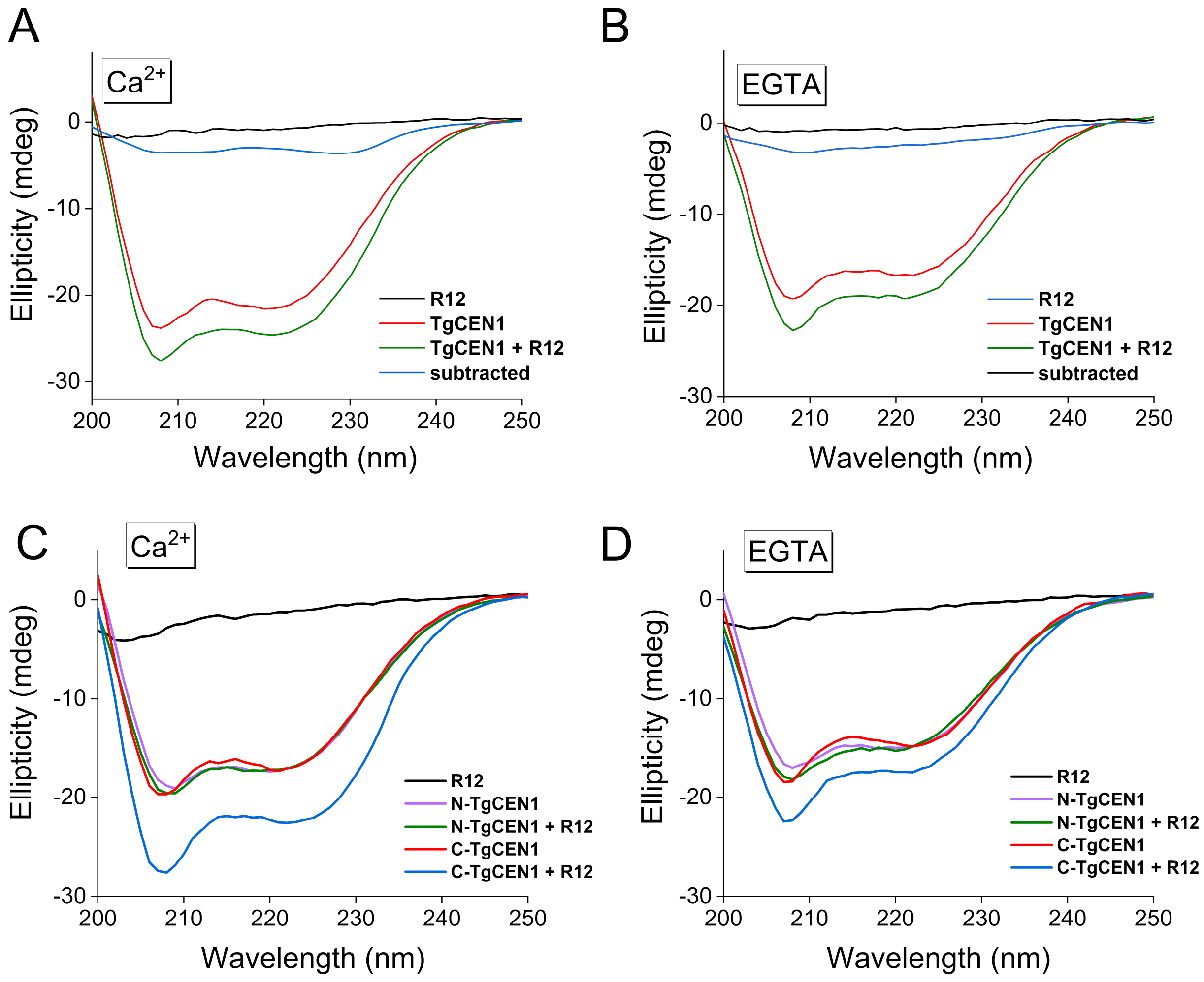

Figure S4. Far-UV CD analysis of R12 binding to intact TgCEN1 and its isolated domains in the presence of $CaCl_2$ or EGTA. (A-B) Far-UV CD spectra of R12 peptide alone (black line), TgCEN1 (red line), and protein-peptide complex (green line) in the presence of (**A**) 5 mM $CaCl_2$ or (**B**) EGTA. The CD spectrum resulting from subtraction of the spectrum of protein-peptide complex from that of protein alone is also shown (blue line). **(C-D)** Far-UV CD spectra of R12 peptide alone (black line), N-TgCEN1 (violet line) or C-TgCEN1 (red line), N-TgCEN1-R12 complex (green line) or C-TgCEN1-R12 complex (blue line) in the presence of (**C**) 5 mM $CaCl_2$ or (**D**) EGTA. In all spectra the same concentration of protein (0.2 mg/mL) was used and a 1:1 molar ratio of R12 peptide was added. Increasing the peptide-to-protein ratio was not accompanied by obvious CD changes, in perfect agreement with the ITC obtained stoichiometry.

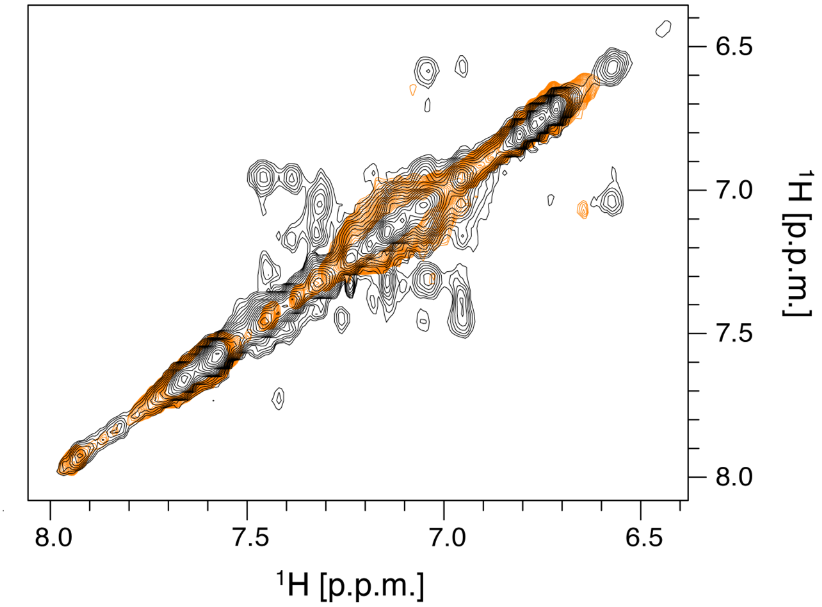

Figure S5. 2D ^{15}N, ^{13}C filtered NOESY spectra of ^{15}N-labeled Ca^{2+}-TgCEN1 (orange) and of unlabeled R12 peptide bound to uniformly ^{15}N-labeled Ca^{2+}-TgCEN1 (black). The increased number of intramolecular ^{1}H-^{1}H NOEs suggests the presence of more ordered regions within the peptide upon binding to the labelled protein.

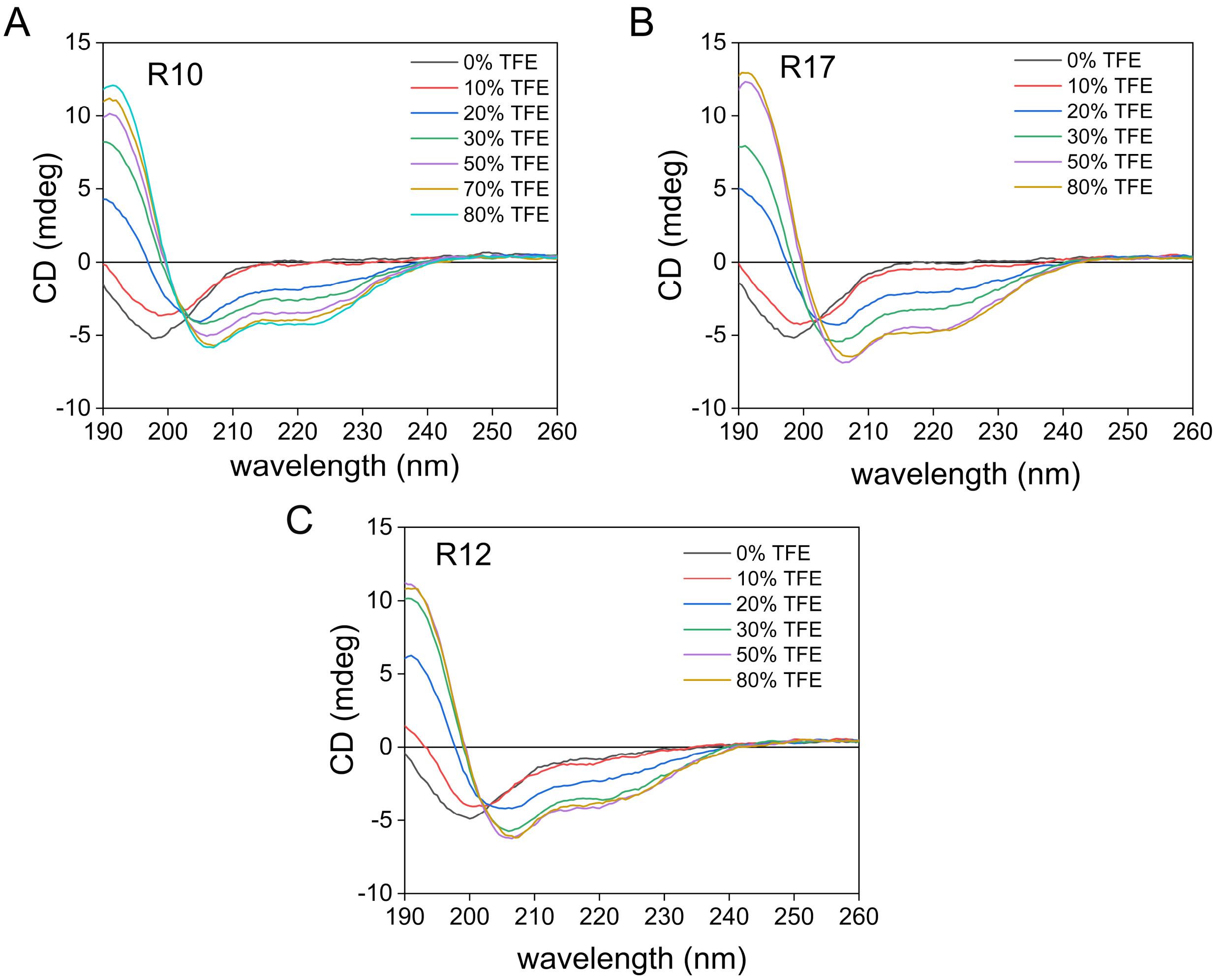

Figure S6. Far-UV CD spectra of 20 µM R10 (A), R17 (B) and R12 (C) peptides in various water/TFE mixtures at 25°C.
